# Statistical profiling of hospital performance using acute coronary syndrome mortality

**DOI:** 10.5830/CVJA-2011-064

**Published:** 2012-11

**Authors:** Samuel OM Manda, Chris P Gale, Alistair S Hall, Mark S Gilthorpe

**Affiliations:** Biostatistics Unit, Medical Research Council, Pretoria, South Africa; Centre for Epidemiology and Biostatistics, University of Leeds, Leeds, United Kingdom; Centre for Epidemiology and Biostatistics, University of Leeds, Leeds, United Kingdom; Centre for Epidemiology and Biostatistics, University of Leeds, Leeds, United Kingdom

**Keywords:** Bayesian methods, health provider performance, league tables

## Abstract

**Background:**

In order to improve the quality of care delivered to patients and to enable patient choice, public reports comparing hospital performances are routinely published. Robust systems of hospital ‘report cards’ on performance monitoring and evaluation are therefore crucial in medical decision-making processes. In particular, such systems should effectively account for and minimise systematic differences with regard to definitions and data quality, care and treatment quality, and ‘case mix’.

**Methods:**

Four methods for assessing hospital performance on mortality outcome measures were considered. The methods included combinations of Bayesian fixed- and random-effects models, and risk-adjusted mortality rate, and rank-based profiling techniques. The methods were empirically compared using 30-day mortality in patients admitted with acute coronary syndrome. Agreement was firstly assessed using median estimates between risk-adjusted mortality rates for a hospital and between ranks associated with a hospital’s risk-adjusted mortality rates. Secondly, assessment of agreement was based on a classification of hospitals into low, normal or high performing using risk-adjusted mortality rates and ranks.

**Results:**

There was poor agreement between the point estimates of risk-adjusted mortality rates, but better agreement between ranks. However, for categorised performance, the observed agreement between the methods’ classification of the hospital performance ranged from 90 to 98%. In only two of the six possible pair-wise comparisons was agreement reasonable, as reflected by a Kappa statistic; it was 0.71 between the methods of identifying outliers with the fixed-effect model and 0.77 with the hierarchical model. In the remaining four pair-wise comparisons, the agreement was, at best, moderate.

**Conclusions:**

Even though the inconsistencies among the studied methods raise questions about which hospitals performed better or worse than others, it seems that the choice of the definition of outlying performance is less critical than that of the statistical approach. Therefore there is a need to find robust systems of ‘regulation’ or ‘performance monitoring’ that are meaningful to health service practitioners and providers.

## Abstract

Incidents of professional failure and the necessity to improve efficiency and quality of care in the health service have led to increasing demand for quality assurance and audits of medical institutions.^1^-^5^ This has allowed quality appraisal and optimal targeting of resources to areas of need. These processes have led to significant improvements in health outcomes; however, variation in hospital performance remains.^5^,^6^

A widely used and acceptable method to control variation in health outcomes is based on case mix adjustment.^7^-^9^ However, failure to adjust appropriately for differences in case mix may result in unfairly targeting hospitals admitting high-risk patients. Indeed, the identification of hospitals having unusual performance depends on the variables used in the risk-adjustment model.^7^,^8^ Furthermore, comparing hospitals on the basis of a risk-adjustment model could be erroneous, as the risk model may be wrong, or suffer from incorrect inclusion of prognostic factors.^4^

More importantly, the disparity in risk-adjusted outcomes may result from a variety of factors including definitions, data quality, structural and institutional management factors, and resource characteristics that have a direct effect on clinical processes.^4^-^6^ To this end, differences in case mix should be accounted for in a suitable risk-adjustment model and differences in definitions and data quality kept to a minimum. Any residual variation in outcome between hospitals would therefore reflect hospital quality of care, the basis for medical institutional profiling methods.^7^-^15^ However the extent to which these hopes are satisfied remains uncertain.

There is a large literature base on statistical methodology for health provider profiling.^10^-^13^ Simple methods use ratios of the observed to the expected outcomes (indirect standardisation) or odds ratios from a logistic regression analysis.^8^,^15^ A number of studies have shown disagreements between different frequentist or Bayesian methods for profiling hospital performance (Marshall and Spiegelhalter,^11^ Austin,^12^ Ohlssen *et al.*,^15^ Delong *et al.*^16^ and Leyland and Boddy^17^). In particular, random-effects models are found to be more conservative in classifying institutions as performance outliers.^11^ There is therefore a need for research to identify statistical models and ways that robustly differentiate between hospitals and remain meaningful to the medical practitioner.^12^

Normand *et al.*,^10^ Marshall and Spiegelhalter,^11^ Austin^12^ and Ohlssen *et al.*^15^ advocated using hierarchical Bayesian random-effects methods for provider profiling. These methods easily permit data pooling across institutions; thus overcoming uncertainty associated with small institutions, which might be outliers by chance alone.^12^ Estimated performances are stabilised and shrunk towards the population average; the degree of shrinkage being larger for small hospitals than for large hospitals.

Bayesian methods provide complete probabilistic information in determining the probability that a hospital-specific risk-adjusted rate exceeds a specified threshold.^11^ Furthermore, a researcher is able to place credible intervals on the derived ranks to quantify the uncertainty associated with institutional ranking before relative performance can be assessed.^11^,^18^

In the current study, rather than calibrate the methods, we concentrated on comparing the performance of four methods and assessing how well they agreed with one another, using Marshall and Spiegelhalter,^11^ and Austin’s approaches.^12^ The methods were applied to data on short-term mortality in acute coronary syndrome (ACS) patients. The data are part of the Myocardial Infarction National Audit Project (MINAP), which currently reports percentage attainment of standards on five clinical process variables, namely door-to-needle and call-to-needle thrombolysis times, and the use of aspirin, beta-blockers and statins post-acute myocardial infarction (AMI).^19^,^20^ A use of the MINAP data for hospital comparison was presented in Gale *et al*.,^5^ using funnel plots on the same five process variables.

To the best of our knowledge, the present study is the first to use an outcome measure and to control for any variation, specifically for case mix, with contemporary data on ACS. We did not duplicate MINAP tabulations or the Gale *et al*.^5^ funnel plot methodology. Instead, we determined (a) whether or not a hospital’s risk-adjusted mortality rate exceeded a specified threshold, and (b) the hospital’s rank, based on its risk-adjusted mortality rate using two statistical models: fixed and hierarchical models, on the number of deaths among patients admitted by the hospital. While this article does not add sufficient new methodological questions on profiling methods, the topic of healthcare performance is timely, important and interesting within the medical and health services domain.

## Methods

MINAP was established in 1998. It is reported to be the largest and most comprehensive clinical database of ACS care in the world and is a valuable resource for monitoring coronary heart disease audit standards for patients presenting with AMI in England and Wales.^20^ All hospitals in England and Wales that treat patients with acute AMI submit data to MINAP. The project collects information on the quality of care and outcome of patients. Each patient entry offers details of the patient’s journey, including the method and timing of admission, in-patient investigations, results and treatment, and, if applicable, dates of death from linkage to the Office of National Statistics, United Kingdom.

Prospective data are collected locally, electronically encrypted and transferred to a central database. The database may be used for identifying performance indicators to identify examples of good practice. With such data, it is feasible to evaluate contemporary care practices consistent with national guidelines for the management of ACS, investigate whether hospital performance varies between hospitals, identify hospital characteristics predictive of adherence to guidelines, and assess whether adherence to guidelines is associated with mortality rates.^7^

We examined all 187 069 ACS events entered into the MINAP database from 1 January 2003 to 31 March 2005. We selected first (index) admissions reported to MINAP and therefore excluded re-admissions. We then analysed all patients who were aged between 18 and 100 years, who had an admission systolic blood pressure between 49 and 250 mmHg, and an admission heart rate between 20 and 200 beats/min.

In total there were 134 hospitals, six of which were discarded from the analyses because of sparse data, i.e. not sufficiently varied (two with one admission, three with fewer than five deaths, one with excessive missing codes on death status). For the remaining 100 686 patients, the overall in-hospital mortality rate was 8.1%, the total mortality rate was 17.8%, and the 30-day mortality rate was 10.2%. Hospital-specific 30-day mortality rates ranged from 5 to 21%, with a median rate of 8.3%.

## Statistical models

We assumed that *O*_i_ is the observed number of 30-day deaths in patients admitted to hospital *i* (*i* = 1, …, 128) and *E*_i_ is the expected number of deaths, given the case mix of its patients. The number of deaths in the period 1 January 2003 to 31 March 2005 can be assumed to follow a Poisson distribution with unknown mean *λ*_i_. Therefore *O*_i_ ~ Poisson (*λ*_i_) taking log *λ*_i_ = log *E*_i_ + θ_i_ where log *E*_i_ is an offset that adjusts for the patient effects and θ_i_ is a residual representing hospital-specific effect of interest.

The expected number *E*_i_ is obtained from a logistic regression on the pooled data, adjusting for relevant risk factors, to determine each patient’s predicted probability of 30-day mortality. These probabilities are then summed within a hospital to give the expected number of deaths at that hospital, given its case mix. The hospital-specific effect θ_i_ is the log-relative risk or logarithm of the hospital’s standardised mortality ratio (log SMR).

Other than to compare hospitals using their SMRs, we used the hospital risk-adjusted 30-day mortality rate (RAMR),^7^ defined as RAMR = µ_30_ exp (θ_i_), where µ_30_ (= 10.2%) is the overall 30-day mortality rate. The RAMR can be thought of as the estimated 30-day mortality rate for a hospital admitting a population of patients identical to the overall case mix.^11^ We adopted Bayesian methods in estimating a hospital-specific random effect θ_i_ to obtain its specific risk-adjusted mortality rate using 10.2 × exp (θ_i_), which we used in this study for institutional profiling.

In order to estimate the hospital-specific effect, we firstly assumed that it has a prior normal distribution with mean 0 and variance 1 000. This is the fixed-effects model, and the prior distribution implies that the hospital-specific standardised mortality rate has a prior mean of 1.

Secondly, as an alternative, we considered a Bayesian random-effects model, which, by using hierarchical modelling, pools data across hospitals. This approach produces more reliable estimates of hospital performance, in that genuinely low or high hospital outliers are identified. It reduces the chance of a small hospital being classified as an outlier by chance alone.^11^

Under the latter modelling approach, it was assumed that the hospital-specific random effects θ_i_ were drawn from a normal distribution with an unknown mean µ_0_ and variance σ_0_^2^. Therefore, θ_i_ ~ Normal (µ_0_, σ_0_^2^), where the hyper-parameters µ_0_ and σ_0_^2^ were the underlying overall log-standardised mortality ratio and between-hospital variance, respectively.

In order to complete the Bayesian implementation of the model, we also needed to specify prior probability distributions for the hyper-parameters µ_0_ and σ_0_^2^ for the hospital-specific random effects, θ_i_ distribution. The hyper-mean, µ_0_ was assigned a normal distribution with mean of 0 and variance 1 000. The hyper-precision, σ_0_^-2^ (inverse of the hyper-variance, σ_0_^2^) was given a gamma distribution with shape and scale parameters both equal to 0.001; implying that the hyper-precision had a mean of 1 and variance 1 000. This prior translates into a locally uniform distribution on the logarithm of the hyper-variance.

We used two ways of identifying outliers; one based on the hospital’s RAMR, and the other based on the rank of RAMR among all the hospitals’ RAMR. Assessments of agreement were initially based on point estimates between each hospital’s ranks, and between risk-adjusted mortality rates. These pairwise agreements could be assessed using Bland–Altman plots.^21^ However, we used simple two-way scatter plots, where agreement was judged against the line of equality.

We concentrated on categorising the different classification outcome measures into low, normal or high mortality risk, and then assessing agreement across the categories. In categorising a hospital’s RAMR, we examined the probability of it exceeding a specified threshold. The overall 30-day mortality rate was 10.2% for our patient cohort. A hospital *i* is classified as a high outlier if Prob [RAMR_i_ > (1 + σ) 10.2] ≥ 0.75 and, similarly, it is classified as a low outlier if Prob [RAMR_i_ < (1 – σ) 10.2] ≥ 0.75, otherwise the hospital is classified as normal.

The threshold value δ can take any value, but values of 10, 15 and 20% are commonly used.^18^ We conservatively chose δ to be 20%, which has the effect of minimising the number of outlying hospitals, therefore hospital *i* is a high outlier if Prob (RAMR_i_ > 12.24) ≥ 0.75, and a low outlier if Prob (RAMR_i_ < 8.16) ≥ 0.75.

For ranks, we calculated Bayesian point estimates and 95% credible intervals of each hospital’s rank. Hospitals whose 95% intervals fell entirely in the bottom or upper quartile of ranks (i.e. upper limit is ≤ 32.75 or lower limit is ≥ 96.25) were classified as low or high outliers, respectively; otherwise they were normally performing hospitals.

With two modelling approaches (the fixed- and random-effects models) plus two ways of classifying hospital performance, we had four different methods for profiling hospitals. In all, there were six possible pair-wise comparisons.

For each comparison, we used the kappa (κ) statistic to assess the amount of agreement between the methods. The statistic measures the proportion of observed-to-expected agreement, and we adopted the convention that κ > 0.75 indicates excellent agreement, κ = 0.4–0.75 indicates good agreement, and κ < 0.4 indicates marginal agreement,^22^ even though κ has been criticised for its limitations. In order to allow for different levels of agreement, we used a weighted κ statistic.

## Implementation

The computation of the models was done using Markov Chain Monte Carlo methods (MCMC); specifically we used Gibbs sampling as implemented in WinBUGS.^23^ For each method considered, three parallel Gibbs sampler chains from independent starting positions were run for 50 000 iterations. We monitored 10 randomly chosen random effects, and for hierarchical models also hyper-parameters for convergence.

Trace plots of sample values of each of these parameters showed that they were converging to the same distribution. We formally assessed convergence of the three chains using Gelman–Rubin reduction factors,^24^ and all were estimated near 1.0 by 15 000 iterations. We therefore took 15 000 iterations to be in the burn-in period. For posterior inference, we used the remaining 35 000 iterations to give a combined sample size of 105 000.

## Results

Existing ACS risk scores include a multitude of factors. Patient age, systolic blood pressure (SBP), heart rate (HR) at admission and ECG findings are systematically included in most of the risk-scoring systems.^25^-^27^ In a large sample of European patients with ACS, age was found to impact on most of the clinical presentations and on hospital mortality.^28^ Therefore inclusion of age in a risk model would account for many of the baseline, prior and clinical risk factors.

The risk variables that we used in the case mix logistic regression model for the risk adjustment are presented in Table 1, where age cut-off points were based on Resengren *et al*.,^28^ and SBP and HR on their fifths. The fitted model had an estimated *c*-statistic (area under the ROC curve) of 0.798, with a 95% confidence interval of 0.794 to 0.803. The inclusion of co-morbidities (e.g. diabetes and chronic renal failure) resulted in loss of data and minor improvement on the *c*-statistics. Using only age, SBP and HR, whether continuous or categorised, resulted in a similar value of the c-statistic of 0.777 (0.772–0.781).

**Table 1.** The Risk-Adjustment Model Of 30-Day Mortality Using Baseline Risk Factors, Discharge ECG Findings And Biochemical Markers

*Risk factor*	*Number of patients*	*Number of deaths (%)*	*Odds ratio (95% CI)*
Age group (years)			
< 55	14 116	233 (1.7)	1.00
55–64	16 396	549 (3.4)	2.02 (1.72–2.37)
65–74	21 442	1 703 (7.9)	5.06 (4.38–5.84)
75–84	23 006	3 656 (15.9)	10.73 (9.33–12.34)
≥ 84	9 249	2 259 (24.4)	18.03 (15.61–20.83)
SBP (mmHg)			
< 117	16 609	3 082 (18.6)	1.00
117–132	16 745	1 716 (10.3)	0.56 (0.52–0.60)
133–146	16 458	1 354 (8.2)	0.43 (0.40–0.46)
147–164	17 072	1 161 (6.8)	0.33 (0.31–0.36)
≥ 165	17 325	1 087 (6.3)	0.27 (0.25–0.29)
Heart rate (beats/min)			
< 62	18 135	1213 (6.7)	1.00
62–72	15 538	991 (6.4)	1.10 (0.99–1.20)
73–83	16 836	1 373 (8.2)	1.38 (0.27–1.51)
84–98	16 600	1 905 (11.5)	1.84 (1.70–2.00)
≥ 99	17 100	2 918 (17.1)	2.55 (2.36–2.75)
Discharge diagnosis			
ST elevation	29 389	3 612 (12.3)	8.59 (6.09–12.11)
Non-ST elevation	29 462	3 379 (11.5)	5.29 (3.75–7.47)
Tropin positive	6 719	368 (5.5)	2.59 (1.81–3.71)
Tropin negative	6 326	58 (0.9)	0.67 (0.43–1.02)
Chest pain	3 136	34 (1.1)	1.00
Other			
Total	84 209	8 400 (9.98)	4.68 (3.29–6.67)

Using this predictive model of 30-day mortality shown in Table 1, we evaluated the expected number of deaths, *E*_i_ in hospital *i* to obtain its standardised mortality ratio, SMR_i_ = *O*_i_ *E*_i_ and risk-adjusted mortality rate, RAMR_i_ = 10.2 × SMR_i_, which ranged from 4.54 to 19.44% with a median of 9.91%.

Table 2 shows the top and bottom five ranked hospitals according to their risk-adjusted 30-day mortality rate. The top or bottom ranked 10 hospitals were more or less the same using only age, SBP and HR but with a slightly longer range, 4.14 to 23.32%.

**Table 2.** Observed, Expected And Risk-Adjusted 30-Day Mortality Rate After ACS Admission, 2003–2005, England And Wales

*Hospital*	Number of admissions*	*Observed deaths*	*Expected deaths*	*RAMR (95% CI)*
Top five				
1	737	39	89.65	4.54 (3.32–6.21)
2	167	5	10.58	4.82 (2.01–11.58)
3	232	9	18.99	4.83 (2.52–9.29)
4	209	10	20.10	5.07 (2.73–9.43)
5	2 158	71	123.56	5.86 (4.64–7.40)
Bottom five				
124	289	42	27.43	15.62 (11.54–21.13)
125	24	5	3.21	15.90 (6.62–38.19)
126	21	4	2.50	16.31 (6.12–43.44)
127	348	63	37.45	17.16 (13.40–21.96)
128	97	19	9.97	19.44 (12.40–30.48)

*With a valid 30-day status.

**Fig. 1:**
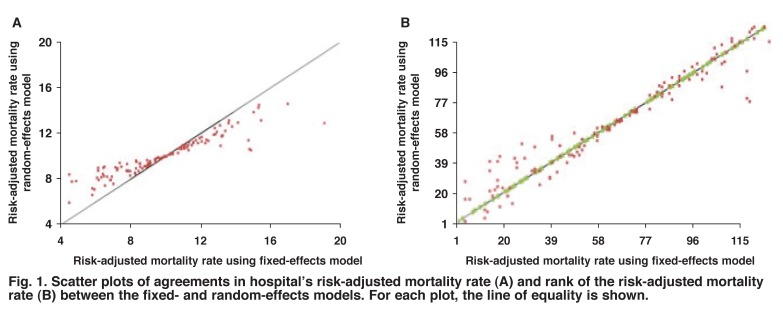
Scatter plots of agreements in hospital’s risk-adjusted mortality rate (A) and rank of the risk-adjusted mortality rate (B) between the fixed- and random-effects models. For each plot, the line of equality is shown.

Comparisons of agreement between a hospital’s risk-adjusted mortality rates and between ranks of the risk-adjusted mortality rates from fitting the fixed- and random-effects models are shown in Fig. 1A, B. For each plot, lines of equality are shown, and comparisons are based on posterior medians. The observed agreement appears to be very poor between the risk-adjusted mortality rates. On the other hand, for the ranks, the points lie evenly around the line of unity, showing very good agreement.

In both plots, agreement is very poor between outcome measures for either low or high outlying hospitals. Furthermore, the plots show that estimated outcome measures are more variable under the fixed-effects model.

The problems observed from using point estimates for assessing agreement can be partially nullified by categorising the hospitals into low, normal and high performing. Comparisons based on categories of risk between different methods are shown in Table 3. All methods were able to classify hospitals as lowand high-outcome outliers; however, only seven and 11 from 128 were classified as such under the hierarchical rank and RAMR methods, respectively, while 31 and 33 were outliers under the fixed-effects rank and RAMR methods, respectively. As expected, profiling methods using hierarchical models were more conservative in classifying hospitals as performance outliers than were the non-hierarchical models.

**Table 3.** Classification Of Hospitals Under The Fixed And Hierarchal Models

	*Fixed RAMR*			*Fixed rank*			*Hierarchical RAMR*		
	*Low*	*Normal*	*High*	*Low*	*Normal*	*High*	*Low*	*Normal*	*High*
Fixed RAMR									
Low	–	–	–	20	0	0	6	14	0
Normal	–	–	–	7	88	0	0	95	0
High	–	–	–	0	9	4	0	8	5
					= 0.71			= 0.46	
Fixed rank									
Low	–	–	–	–	–	–	6	21	0
Normal	–	–	–	–	–	–	0	96	1
High	–	–	–	–	–	–	0	0	4
								= 0.44	
Hierarchical rank									
Low	2	0	0	2	0	0	2	0	0
Normal	18	95	8	25	96	0	4	117	0
High	0	0	5	0	1	4	0	0	5
		= 0.32			= 0.29			= 0.77	

The observed agreement in the methods’ classification of hospitals ranged from 90 to 98% of the time, the highest being between the two hierarchical methods. In only one of the six comparisons was agreement excellent, as reflected by the κ statistic of 0.77. In three cases, the agreement was moderate (0.40 < κ < 0.75). In the remaining two cases, the agreement was only marginal (κ = 0.29–0.32), and these involved comparisons of the random-effects rank and fixed-effects methods. The cross tabulations in Table 3 are in close agreement with those obtained when using only age, SBP and HR in the risk-adjustment model, an indication that our results are insensitive to which factors are included in the risk-adjustment model.

The results presented here are based on arbitrary choices. In particular, the prior for the between-hospital variation is critical as it dictates how much shrinkage is assumed in the individual hospital estimates.^29^ However, there is no standard solution to the problem of choosing a prior on the random-effects variance in hierarchical models. In standard Bayesian analyses, the inverse-gamma prior family is preferred because of its conditional conjugate properties, which allows ease of mathematical derivations. But this prior has been shown to give biased results.^30^

On the other hand, the threshold values for RAMR have an influence of the number of hospitals classified as outliers. We performed a limited-sensitivity analysis to find out the extent to which the choices impact on the results. We used a uniform (0, 100) prior on the random-effects standard deviation σ_o_ and 15% for the threshold value δ. The uniform prior produced exactly the same classifications of the hospitals as the inverse-gamma prior on the random-effects variance. Using a threshold of 15% affected only the 117 hospitals that were previously classified as normal, and now two were classified as low outliers and five as high outliers. Our results were therefore not affected by changes in random-effects variances but slightly so when the threshold value was changed.

## Discussion

This study compared the performance of four methods for profiling hospitals and assessed their agreement. The methods included combinations of two Bayesian methods, fixed and hierarchical, and two ways of identifying outliers, rank and exceeding some threshold using a hospital’s risk-adjusted mortality rate; two were based on a hospital’s rank for its risk-adjusted mortality rate, obtained from fitting both fixed- and random-effects models. The agreements between the different methods were empirically examined using an extensive dataset of ACS patients.

Even though all the methods were able to classify hospitals as low- and high-outcome outliers, profiling methods using random-effects models were more conservative than fixed-effects models in classifying hospitals as having better- or worse-than-expected mortality. These findings were expected on theoretical grounds and support the results from a multitude of prior studies, showing that random-effects models identify fewer performance outliers.^8^,^11^

In the present study, the observed agreement in the methods’ classification of hospitals ranged from 90 to 98%, the highest being between the methods within each effects model. The agreement was excellent (κ = 0.77) in only one of the six comparisons. Otherwise, in all the remaining five scenarios, the agreement was, at best moderate (κ < 0.75).

Our findings relied on routinely collected clinical data. These types of data suffer from incompleteness and inaccuracy of the variables entered.^31^ In our preliminary investigation, 11% of the total patients had missing codes on survival status. We did not have full data for admission age, SBP, HR, ECG findings and biochemical markers of the patients. Other risk variables that may have been used also demonstrated missing data, thus limiting the number of risk factors in the case mix adjustment model on this occasion. However, our findings were shown to be robust to which factors were included in the risk-adjustment model. Indeed, difficult-to-obtain key clinical variables add little to the predictive power of ACS risk scores.^27^

It may well be that the hospital performance variation exhibited in this study was substantially contributed to by the variation in definitions and data quality, as alluded to by Lilford *et al*.^4^ However, it is unlikely that these issues alone could be attributed to the outcome variation found across the four analytical strategies examined.

We did not impute for missing data since other researchers have shown that this does not affect the prediction model or mortality.^32^ A more elaborate assessment of MINAP data quality and validity on the resulting classification of hospitals is the subject of a British Heart Foundation-funded project within our group undertaken by Gale *et al*.^33^ For the present study, it suffices to say that the number of patients analysed and the data used were of sufficient quality to enable a comparison of different methods to assess the hospitals’ performance for 30-day mortality among ACS patients. However, we remain cautious regarding the exact inference made for some hospitals, given their data quality.

We performed a limited-sensitivity analysis to different prior specifications of the hospital random-effects variation and threshold values. We found classification of outlying hospitals was not affected by changes in the random-effects variations, but it was slightly affected when the thresholds were changed.

A more elaborate sensitivity analysis would alter specification of the hospital random-effects distribution as the assumed normal distribution is not robust and flexible enough to account for outlying hospital effects. Therefore it may be necessary in future research to model the hospital effects more flexibly, for example by heavy-tailed *t* distributions to investigate both sensitivity and robustness of the results, as in Manda,^34^ or mixtures or non-parametric Dirichlet distributions, as in Ohlssen.^35^

The threshold level chosen and the required probability of exceeding this threshold to classify a hospital using the risk-adjusted mortality rate as an outlier were subjective and completely arbitrary. We could have used other thresholds and probabilities, as in Austin,^12^ which may have generated stronger or weaker levels of agreement between the methods. Furthermore, the requirement that intervals of the ranks must lie entirely in the bottom or top quarters of ranks for the hospital to be classified as an outlier was also arbitrary but has been used before.^11^,^12^

Results from any study on profiling hospitals’ performance are predictably used to produce league tables of performance. We are aware of the many criticisms surrounding the statistics used in measuring performance and the subsequent ranking of hospitals. We did not intend to contribute to this controversy. Our aim was to describe and compare the performance of four different Bayesian methods for institutional profiling. In using ranks to compare hospitals, caution should be exercised since most hospitals had considerably overlapping intervals, which made it difficult to obtain reliable ranking, especially for hospitals admitting fewer patients.

We follow Normand *et al*.,^10^ Marshall and Spiegelhalter,^11^ Austin^12^ and Ohlssen *et al*.^18^ in advocating the use of Bayesian methods, which when pooling data across hospitals, handles the problem of small hospitals better than frequentist methods, for which a minimum number of patients is required before a hospital can be included.^12^ However, if we are willing to accept wide confidence intervals, the exact probabilistic methods can be used within a frequentist framework to handle small hospitals (see Luft and Brown^36^). Furthermore, it is much easier within Bayesian methods to determine uncertainty associated with the ranks, which are very sensitive to sampling variations (see Marshall and Spiegelhalter^11^ and Greenwood^18^).

The main interest of this work was not to find the best model for hospital profiling, but to investigate whether or not the methods agree. In order to inform which method gives a better fit would require other model-checks statistics, such as posterior predictive checks.

## Conclusion

The main overall finding from our example is that the choice of ways to classify a hospital is less critical than the statistical method used. We suggest profiling hospitals using a hierarchical model and RAMR with an appropriate threshold, which seems to offer more reliable results. However, these methods warrant further investigation, possibly of simulated data sets in which the impact of underlying assumptions (and derivation thereof) may be evaluated. There is a need for robust systems of ‘regulation’ or ‘performance monitoring’, which, with more rigorous work, we hope to achieve in the future.
